# Log-linear relationship between endogenous insulin secretion and glycemic variability in patients with type 2 diabetes on continuous glucose monitoring

**DOI:** 10.1038/s41598-021-88749-9

**Published:** 2021-04-27

**Authors:** Aika Miya, Akinobu Nakamura, Takahisa Handa, Hiroshi Nomoto, Hiraku Kameda, Kyu Yong Cho, So Nagai, Yoichi M. Ito, Hideaki Miyoshi, Tatsuya Atsumi

**Affiliations:** 1 Department of Rheumatology, Endocrinology and Nephrology, Faculty of Medicine and Graduate School of Medicine, Hokkaido University, N-15, W-7, Kita-ku, Sapporo, 060-8638 Japan; 2 Division of Diabetes and Endocrinology, Department of Medicine, NTT Sapporo Medical Center, Sapporo, Japan; 3 Clinical Research and Medical Innovation Center, Hokkaido University Hospital, Sapporo, Japan; 4 Biostatistics Division, Clinical Research and Medical Innovation Center, Hokkaido University Hospital, Sapporo, Japan; 5 Division of Diabetes and Obesity, Faculty of Medicine and Graduate School of Medicine, Hokkaido University, Sapporo, Japan

**Keywords:** Type 2 diabetes, Endocrine system and metabolic diseases, Diabetes complications

## Abstract

The contribution of endogenous insulin secretion to glycemic variability (GV) may differ between patients with impaired insulin secretion and those with preserved secretion. Our objective was to determine the linearity of the relationship between fasting C-peptide (CPR) as a marker of endogenous insulin secretion and GV in type 2 diabetes (T2DM), regardless of the type of antidiabetic treatment. We conducted a prospective observational study using continuous glucose monitoring obtained from 284 Japanese outpatients with T2DM with various HbA1c values and antidiabetic treatment. We constructed a prediction curve of base-line CPR *versus* coefficient of variation (CV) and identified the clinical factors associated with CV using multiple regression analysis. Fasting CPR showed a significant negative log-linear relationship with CV (*P* < 0.0001), and the latter being strikingly high in the low-CPR group. The multiple regression analysis showed that low CPR was an independent predictor of high CV (*P* < 0.0001). The significant correlations were sustained in both patients with/without insulin treatment. The contribution of endogenous insulin secretion to GV depends on the extent of insulin secretion impairment. Fasting CPR may represent a useful indicator of GV instability in T2DM.

## Introduction

The primary objective of diabetes treatment is to help patients live without the anxiety associated with the risk of hypoglycemia and diabetes-related complications^1^. Continuous glucose monitoring (CGM) is able to track the changes in glucose concentration throughout the day and permits the assessment of glycemic variability (GV)^2^. CGM-based studies have shown that GV instability is not only associated with higher risks of hypo- and hyperglycemia^3^, but also of the onset and progression of diabetes-related microvascular^4–6^, and macrovascular complications^7, 8^. Therefore, antidiabetic treatment strategies should be aimed at reducing GV. To achieve this target, it is necessary to clarify the patient factors that influence GV. In type 1 diabetes, low endogenous insulin secretion is associated with GV instability^9^. In addition, we have previously shown that impaired endogenous insulin secretion affects GV stability in type 2 diabetes^10^. However, other previous studies of type 2 diabetes have shown the inconsistent results regarding the relationship between endogenous insulin secretion and GV, including a lack of relationship between fasting C-peptide (CPR) concentration and GV in type 2 diabetes^11–13^. These previous studies were conducted in patients with preserved insulin secretion. To explain such heterogeneity in the correlation, comprehensive data regarding GV in type 2 diabetes using CGM, including in patients with severe insulin secretion disorders, were to be interpreted. Such investigations may demonstrate that the contribution of endogenous insulin secretion to GV differs between patients with impaired insulin secretion and those with preserved secretion. Therefore, we hypothesized that the relationship between endogenous insulin secretion and GV is not linear, but instead log-linear.

In the present study, we used CGM to investigate whether endogenous insulin secretion, evaluated using fasting CPR, has a log-linear relationship with GV in type 2 diabetes, including those with severely impaired endogenous insulin secretion^10^. Furthermore, we explored whether fasting CPR concentration could be a predictor of GV instability, regardless of the type of antidiabetic treatment used in those patients.

## Methods

### Study population and design

We conducted a prospective observational study. Japanese outpatients with type 2 diabetes were recruited between April 2018 and September 2019 at four medical institutions, as described previously^10^. Briefly, patients of ≥ 20 years of age were eligible if they consented to undergo ambulatory CGM, regardless of their HbA1c level, sex, duration of diabetes, or the presence of complications of diabetes. The exclusion criteria were as follows: (1) type 1 diabetes meeting the diagnostic criteria^14^, (2) hospitalization within the preceding 3 months, (3) diabetic ketosis/coma, (4) serious infection, (5) recent or planned surgery, (6) trauma within the preceding 6 months, (7) current glucocorticoid therapy, (8) difficulty with dietary intake, or (9) pregnancy or lactation. CGM data, fasting blood samples, and clinical information (age, sex, anthropometric measurements, duration of diabetes in years, treatment regimen, and medical history) were obtained from each participant.

The study was registered with the University Hospital Medical Information Network Center (registration number UMIN 000029993). The study protocol was approved by the Institutional Review Board of Hokkaido University Hospital Clinical Research and Medical Innovation Center (017-0147), and the study was conducted in accordance with the Declaration of Helsinki. Written informed consent was obtained from all the patients.

### Biochemical analyses and data collection

Ambulatory CGM (using a FreeStyle Libre Pro sensor; Abbott Diabetes Care, Alameda, CA, USA) was performed for 14 consecutive days. The CGM data remained blinded for patients and physicians because the CGM system used was a professional version that permitted blinded CGM. Thus, patients were not able to access any real time information on their GV that might have influenced their lifestyle behaviors, including their dietary choices, during the study. After the monitoring period, we excluded data collected during the first and last days of wearing the device, considering possible inaccuracies relating to its attachment and removal^15^. We analyzed the CGM data for the remaining period using GlyCulator2 software^16^. Because an international consensus statement recommends the coefficient of variation (CV) as the primary measure of GV^17^, we analyzed the CV as an index of GV using the following formula: 100 × [SD of glucose]/[mean glucose]. The standard deviation (SD) glucose concentration, the mean amplitude of glycemic excursions (MAGE)^18^, and the mean glucose concentration were also analyzed as secondary indices of GV. The low blood glucose index (LBGI) and high blood glucose index (HBGI), which increase with the frequency and extent of hypoglycemia and hyperglycemia, respectively, were also analyzed^19^. Furthermore, we calculated three key CGM-related indices: the percentage of readings and time per day within the target glucose range (TIR: 3.9–10.0 mmol/L), time below target glucose range (TBR: < 3.9 mmol/L), and time above the target glucose range (TAR: > 10.0 mmol/L), as recommended by the international consensus statement^20^.

Blood samples were collected after an overnight fast. Fasting plasma glucose (FPG), CPR, HbA1c, and estimated glomerular filtration rate (eGFR) were measured using standard techniques. The body weight and height of the patients were measured using a calibrated scale. Body mass index (BMI) was calculated as body weight (in kg) divided by height (in m^2^). Other data, including age, sex, the diabetes medications being used, and medical history were collected using a questionnaire that was administered by the attending physicians.

### Data analysis

To define the relationship between endogenous insulin secretion and GV instability, we constructed a scatter plot of fasting CPR *versus* CV. The prediction formula for CPR *versus* CV was estimated after both CPR and CV were logarithmically transformed. The clinical factors related to GV, including CPR, were determined after CPR was logarithmically transformed. Spearman rank-order correlation was used to determine the relationships between continuous variables, such as age. Bivariate analyses of CGM-based metrics of GV and categorical variables, such as sex, were performed using the Mann–Whitney test. The results are shown as the median (interquartile range). Multiple regression analysis was performed using variables identified in previously published research^11^ and those that were significant (*P* < 0.05) in the univariate analysis. We have demonstrated that these items had linear relationships with CGM-based metrics of GV (Supplementary Figs. S1, S2).

To further evaluate the contribution of CPR to CV, the patients were allocated to three subgroups according to whether they had low (CPR < 1 ng/mL), moderate (1 ng/mL ≤ CPR < 2 ng/mL), or high (CPR ≥ 2 ng/mL) CPR concentrations, based on our prediction curve for CPR *versus* CV. Then, the biochemical and anthropometric characteristics of the three subgroups were compared using one-way analysis of variance, the chi-square test, or the Kruskal–Wallis test, as appropriate. Because the international consensus statement defines stable GV using a CV < 36% and unstable GV using a CV ≥ 36%^17^, the number of patients with CV ≥ 36% was compared among the three subgroups using the CV distribution and the chi-square test. Next, to determine whether insulin and antidiabetic treatment use modified the association of interest, the patients were allocated to two subgroups according to whether they received insulin and antidiabetic treatments or not. Additionally, to investigate whether the use of a basal-only or basal-bolus insulin regimen modified the association of interest, we allocated the patients on insulin to two subgroups, according to their insulin regimen, and a similar stratified analysis was carried out.

All the statistical tests performed were two-sided, and *P* < 0.05 was considered to represent statistical significance. Statistical analyses were performed using JMP Pro 14.0.0 (SAS Inc., Cary, NC, USA).

## Results

### Patients characteristics

Of a total of 311 patients enrolled in the study, 27 were excluded because they met one or more of the exclusion criteria described previously^10^. The remaining 284 patients (123 women) were included in the subsequent analyses. Their characteristics are shown in Table 1. The overall median age of the patients was 68 years, their median BMI 25.0 kg/m^2^, and their median duration of diabetes 14 years. The median CPR concentration was 1.7 ng/mL and the median CV was recorded as 27.8.

**Table 1:** Characteristics of the participants.

	Value for the full cohort
*n*	284
Age (years)	68.0 (59.0, 76.0)
Number of women, n (%)	123 (43.3)
BMI (kg/m^2^)	25.0 (22.6, 27.9)
Duration of diabetes (years)	14 (8, 22)
**Diabetes treatment**	
Insulin use, n (%)	120 (42.3)
Basal-only regimen, n (%)	65 (22.9)
Basal-bolus regimen, n (%)	55 (19.4)
Use of sulfonylurea, n (%)	74 (26.1)
Use of glinide, n (%)	33 (11.6)
Use of Metformin, n (%)	173 (60.9)
Use of Thiazolidine, n (%)	19 (6.7)
Use of sodium-glucose cotransporter 2 inhibitor, n (%)	72 (25.4)
Use of α-GI, n (%)	43 (15.1)
Use of DPP-4 inhibitor, n (%)	194 (68.3)
Use of glucagon-like peptide-1 receptor agonist, n (%)	40 (14.1)
FPG (mg/dL)	137.0 (119.3, 157.5)
HbA1c (%)	7.1 (6.7, 7.8)
HbA1c (mmol/mol)	54 (50, 61)
CPR (ng/mL)	1.7 (1.1, 2.5)
CPR (nmol/L)	0.6 (0.4, 0.8)
eGFR (mL/min/1.73m^2^)	66.0 (53.3, 79.5)
24-h mean Glucose (mg/dL)	146.2 (129.0, 166.3)
CV	27.8 (23.7, 32.5)
SD (mg/dL)	40.3 (33.2, 51.4)
MAGE	105.4 (87.5, 134.0)
LBGI	0.3 (0.1, 0.9)
HGBI	4.1 (2.5, 7.0)
TBR (%)	0.1 (0, 2.1)
TIR (%)	76.9 (63.7, 87.4)
TAR (%)	20.2 (10.6, 33.8)

Values are expressed as median (interquartile range), or number (%) of participants in each category.

*BMI* body mass index, *α-GI* alpha-glucosidase inhibitor, *DPP-4* dipeptidyl peptidase-4, *FPG* fasting plasma glucose, *CPR* C-peptide, *eGFR* estimated glomerular filtration rate, *CV* coefficient of variation, *SD* standard deviation, *MAGE* mean amplitude of glycemic excursions, *LBGI* low blood glucose index, *HBGI* high blood glucose index, *TBR* percentage of time below target glucose range, *TIR* percentage of time within target glucose range, *TAR* percentage of time above target glucose range.

### A prediction curve of baseline C-peptide versus glucose variability

**Figure 1:**
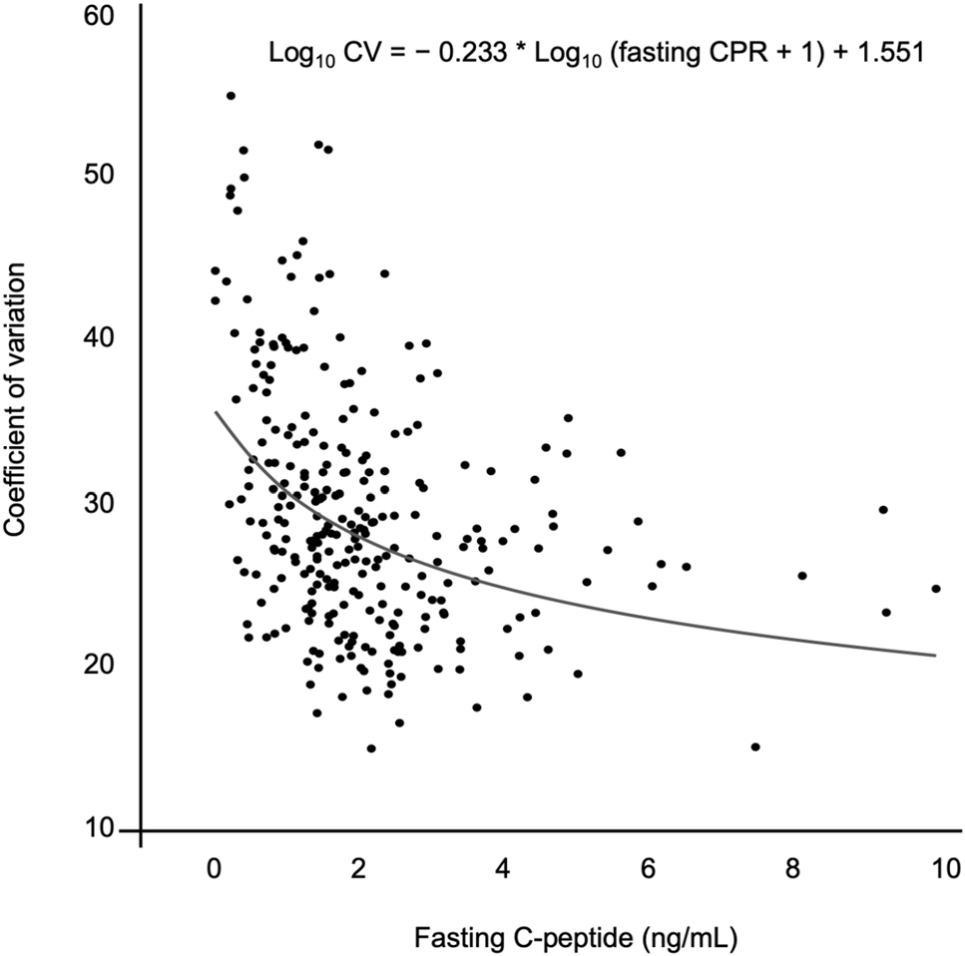
Scatter plot and prediction curve for fasting C-peptide *versus* coefficient of variation (n = 284).

We first constructed a scatter plot of CPR *versus* CV to visualize the potential relationship between these two parameters. For a better understanding of the results, Fig. 1 shows the log-linear relationship between CPR and CV. The prediction curve, constructed using this scatter plot, showed a marked increase in CV with low CPR and relatively low CV with high CPR. CV had significant negative correlation with CPR (ρ =  − 0.39, *P* < 0.0001). The prediction formula was estimated to be the following Eq. (1).

1 $${\text{Log}}_{{{1}0}} {\text{CV }} = \, -\, 0.{233 } \times {\text{ Log}}_{{{1}0}} \left( {{\text{fasting CPR }} + { 1}} \right) \, + { 1}.{551,}$$

CV also showed significant negative correlation with BMI, FPG, and eGFR (Table 2). In addition, CV had significant positive correlation with the duration of diabetes. The CV in patients on insulin was significantly greater than that of non-insulin patients. The CV in patients treated with an alpha-glucosidase inhibitor (α-GI) or dipeptidyl peptidase-4 (DPP-4) inhibitor was significantly smaller than in patients not treated with these drugs. No relationships were identified between CV and other factors, such as HbA1c, age, and other antidiabetic treatments. We assessed the multicollinearity in the variables shown to be associated with CV using Spearman’s correlation analysis (Supplementary Table S1). There was a relatively strong correlation between CPR and BMI, with a correlation coefficient of 0.51. The absolute values of the correlation coefficient for other variables were lower. The variance inflation factor (VIF) for these parameters was less than 2.0 (Supplementary Table S2). Thus, there were no indications of multicollinearity between these variables. Low CPR was an independent predictive marker for high CV, according to multiple regression analysis (regression coefficient (β): − 0.285, 95% confidence interval [CI] − 0.234 to − 0.092, *P* < 0.0001). Insulin use, non-use of an α-GI or DPP-4 inhibitor, and low eGFR were also predictors of high CV (Table 3). We also performed the calculation after correcting CPR for the concomitant plasma glucose level using CPR index, calculated using the formula: 100 × fasting CPR (ng/mL)/FPG (mg/dL)^21^. The findings were similar to those obtained using the fasting CPR (data not shown).

**Table 2:** Correlations between CV and clinical factors.

	ρ	*P* value
Age	0.08	0.1558
Sex (men, women)*	(27.8, 27.8)	0.9709
BMI	− 0.24	< 0.0001
Duration of diabetes	0.24	< 0.0001
Insulin use (yes, no)*	(30.6, 26.1)	< 0.0001
Use of sulfonylurea (yes, no)*	(28.6, 27.5)	0.5752
Use of glinide (yes, no)*	(29.2, 27.6)	0.1960
Use of Metformin (yes, no)*	(27.8, 27.8)	0.8491
Use of Thiazolidine (yes, no)*	(27.5, 27.8)	0.9343
Use of sodium-glucose cotransporter 2 inhibitor (yes, no)*	(26.8, 28.2)	0.1074
Use of α-GI (yes, no)*	(24.7, 28.2)	0.0021
Use of DPP-4 inhibitor (yes, no)*	(27.0, 29.0)	0.0202
Use of glucagon-like peptide-1 receptor agonist (yes, no)*	(29.0, 27.5)	0.1002
FPG	− 0.21	0.0003
HbA1c	0.06	0.3063
CPR	− 0.39	< 0.0001
eGFR	− 0.14	0.0178

Spearman rank-order correlation was used to determine the strength of the relationships.

*CV* coefficient of variation, *BMI* body mass index, *α-GI* alpha-glucosidase inhibitor, *DPP-4* dipeptidyl peptidase-4, *FPG* fasting plasma glucose, *CPR* C-peptide, *eGFR* estimated glomerular filtration rate.

*The Mann–Whitney test was used for bivariate analysis of the relationship between CV and the clinical factor. The results are median CV.

**Table 3:** Relationships between clinically relevant factors and log-transformed CV, according to multiple regression analysis.

	β	95% CI	*P* value
BMI (kg/m^2^)	− 0.091	− 0.005 to 0.001	0.1143
Duration of diabetes (years)	0.109	− 0.000 to 0.002	0.0514
Insulin use	0.230	0.013 to 0.036	< 0.0001
Use of α-GI	− 0.205	− 0.045 to − 0.015	< 0.0001
Use of DPP-4 inhibitor	− 0.133	− 0.026 to − 0.004	0.0086
FPG (mg/dL)	− 0.043	− 0.000 to 0.000	0.4242
CPR (log ng/mL)	− 0.285	− 0.234 to − 0.092	< 0.0001
eGFR (mL/min/1.73 m^2^)	− 0.135	− 0.001 to − 0.000	0.0144

*β* regression coefficient, *95% CI* 95% confidence interval, *CV* coefficient of variation, *BMI* body mass index, *α-GI* alpha-glucosidase inhibitor, *DPP-4* dipeptidyl peptidase-4, *FPG* fasting plasma glucose, *CPR* C-peptide, *eGFR* estimated glomerular filtration rate.

### Relationship between baseline C-peptide and hypo- or hyperglycemia

A log-linear relationship between CPR and LBGI was observed. LBGI showed a significant negative correlation with CPR (ρ =  − 0.25, *P* < 0.0001) as well as the relationship between CPR and CV. As shown in Supplementary Table S3, LBGI also exhibited a significant negative correlation with BMI, FPG, HbA1c, and eGFR. The LBGI in patients treated with insulin was significantly greater than that in patients not treated with insulin. No relationships were identified between the LBGI and other factors. According to multiple regression analysis, low CPR was an independent predictive marker for a high LBGI (β: − 0.163, 95% CI − 0.327 to − 0.043, *P* = 0.0107). Insulin use, low FPG, low HbA1c, and low eGFR were also predictors of a high LBGI (Supplementary Table S4). In contrast, a HBGI was not significantly correlated with CPR (ρ =  − 0.11, *P* = 0.0742).

### The contribution of C-peptide to coefficient of variation

**Figure 2:**
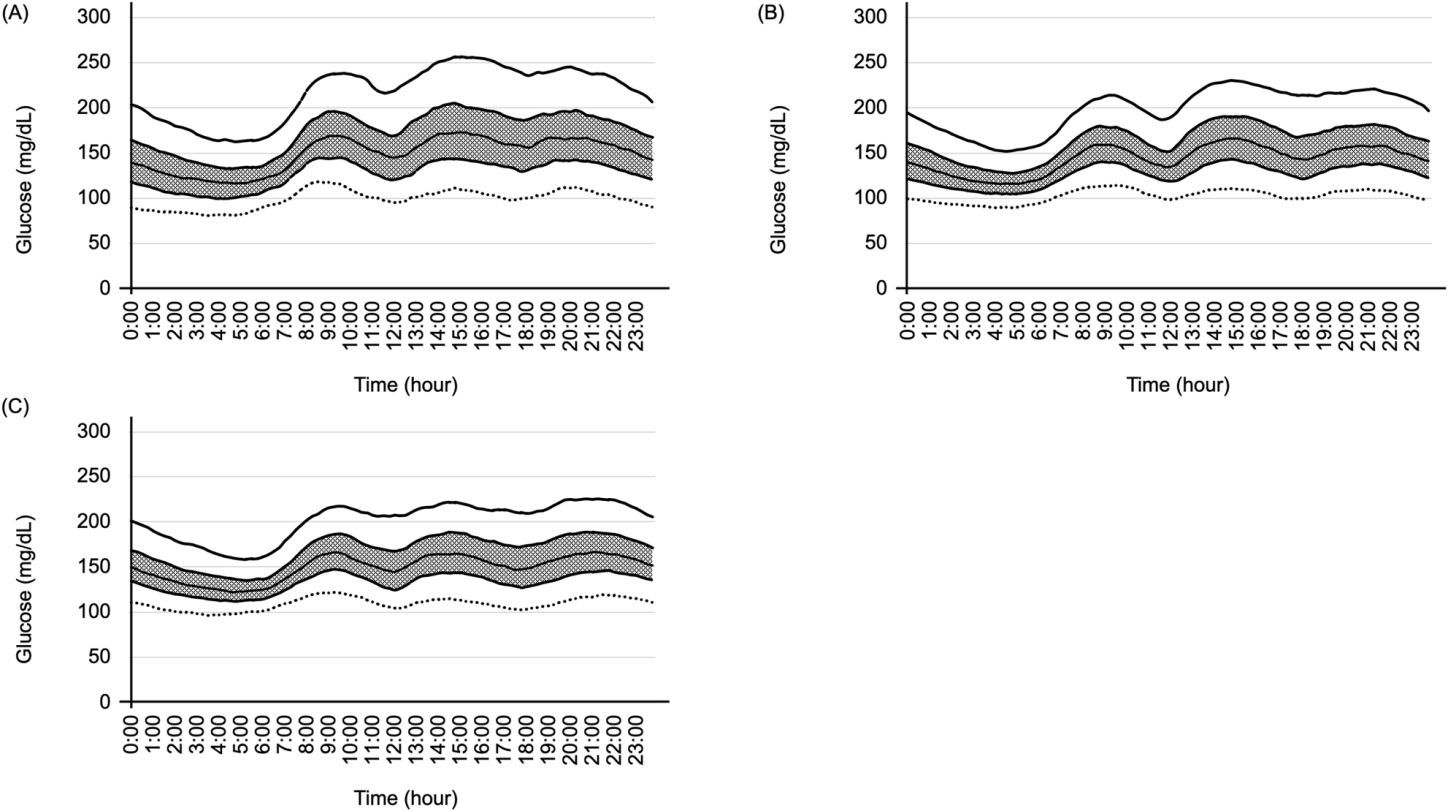
Mean glucose profile of patients in the three C-peptide subgroups: (**A**) low C-peptide (CPR < 1 ng/mL, n = 62), (**B**) moderate C-peptide (1 ng/mL ≤ CPR < 2 ng/mL, n = 113) and (**C**) high C-peptide (CPR ≥ 2 ng/mL, n = 109). Smoothed curves represent the 10th (fine dotted line), 25th (dotted line), median (50th, solid line), 75th (broken line), and 90th (thick dotted line) frequency percentiles. Unstable glucose variability appears to be present in the low C-peptide subgroup.

The 284 patients were allocated to three subgroups: low CPR (CPR < 1 ng/mL, n = 62), moderate CPR (1 ng/mL ≤ CPR < 2 ng/mL, n = 113), and high CPR (CPR ≥ 2 ng/mL, n = 109). The biochemical and anthropometric characteristics of each group are shown in Table 4. Age, BMI, the duration of diabetes, the type of antidiabetic treatment, and FPG were associated with the CPR concentration. The indices of GV (CV, SD, and MAGE), and the key CGM indices (LBGI, HBGI, TBR, TIR, and TAR), were also associated with the CPR concentration. The mean glucose profiles of the patients in each of the three CPR groups are shown in Fig. 2. In summary, the GV appeared to be more unstable in the low CPR group than in the moderate and high CPR groups. Supplementary Figure S3 shows the CV distribution among the three subgroups. The number of patients with CV ≥ 36%, defined as unstable GV, was significantly higher in the low CPR subgroup than in the medium and high CPR subgroups (Table 4).

**Table 4:** Characteristics of the patients, according to their CPR levels.

	Degree of CPR			*P* value
	Low CPR group	Moderate CPR group	High CPR group	
*n*	62	113	109	
Age (years)	72.0 (66.0, 78.0)	68.0 (58.5, 75.0)	66.0 (56.5, 73.5)	0.0132
Number of women, n (%)	28 (45.2)	50 (44.3)	45 (41.3)	0.8568
BMI (kg/m^2^)	22.3 (20.3, 24.4)	24.8 (22.4, 26.7)	27.1 (24.7, 30.1)	< 0.0001
Duration of diabetes (years)	17 (13, 24)	14 (8, 23)	11 (6, 20)	0.0021
**Diabetes treatment**				
Insulin use, n (%)	44 (71.0)	45 (39.8)	31 (28.4)	< 0.0001
Basal-only regimen, n (%)	20 (32.3)	23 (20.4)	22 (20.2)	0.1390
Basal-bolus regimen, n (%)	24 (38.7)	22 (19.5)	9 (8.3)	< 0.0001
Use of sulfonylurea, n (%)	12 (19.4)	33 (29.2)	29 (26.6)	0.3600
Use of glinide, n (%)	8 (12.9)	14 (12.4)	11 (10.1)	0.8137
Use of Metformin, n (%)	30 (48.4)	74 (65.5)	69 (63.3)	0.0692
Use of Thiazolidine, n (%)	2 (3.2)	12 (10.6)	5 (4.6)	0.0926
Use of sodium-glucose cotransporter 2 inhibitor, n (%)	7 (11.3)	28 (24.8)	37 (33.9)	0.0046
Use of α-GI, n (%)	11 (17.7)	20 (17.7)	12 (11.0)	0.3088
Use of DPP-4 inhibitor, n (%)	40 (64.5)	76 (67.3)	78 (71.6)	0.6060
Use of glucagon-like peptide-1 receptor agonist, n (%)	6 (9.7)	12 (10.6)	22 (20.2)	0.0650
FPG (mg/dL)	128.0 (106.8, 147.5)	137.0 (118.5, 153.0)	144.0 (125.0, 166.5)	0.0002
HbA1c (%)	7.2 (6.7, 7.8)	7.1 (6.6, 7.7)	7.1 (6.8, 7.8)	0.4051
HbA1c (mmol/mol)	55 (49, 61)	54 (48, 60)	54 (50, 61)	0.4051
eGFR	66.0 (52.8, 76.8)	67.0 (55.7, 82.4)	64.0 (44.9, 79.4)	0.2456
24-h mean glucose (mg/dL)	147.1 (134.0, 168.5)	141.9 (127.4, 157.7)	148.6 (126.1, 168.5)	0.2941
CV	32.8 (27.7, 39.4)	27.8 (24.5, 32.0)	25.9 (21.9, 29.0)	< 0.0001
CV ≥ 36, n (%)	26 (41.9)	16 (14.2)	5 (4.6)	< 0.0001
SD (mg/dL)	50.0 (41.5, 63.7)	39.1 (33.4, 47.4)	38.0 (31.2, 48.8)	< 0.0001
MAGE	131.6 (103.8, 156.9)	102.7 (85.0, 130.0)	96.8 (77.6, 127.5)	< 0.0001
LBGI	0.6 (0.2, 1.2)	0.2 (0.1, 0.9)	0.1 (0.0, 0.6)	0.0001
HGBI	5.5 (3.2, 8.2)	3.6 (2.5, 5.7)	4.1 (2.1, 7.0)	0.0386
TBR ≥ 4%, n (%)	17 (27.4)	17 (15.0)	11 (10.1)	0.0112
TIR ≤ 70%, n (%)	33 (53.2)	33 (29.2)	35 (32.1)	0.0041
TAR ≥ 25%, n (%)	33 (53.2)	34 (30.1)	48 (44.0)	0.0074

Values are expressed as median (interquartile range), or number (%) of patients in each category.

One-way analysis of variance, the Kruskal–Wallis test, or the chi-square test was used to compare the three groups.

*CPR* C-peptide, *BMI* body mass index, *α-GI* alpha-glucosidase inhibitor, *DPP-4* dipeptidyl peptidase-4, *FPG* fasting plasma glucose, *eGFR* estimated glomerular filtration rate, *CV* coefficient of variation, *SD* standard deviation, *MAGE* mean amplitude of glycemic excursions, *LBGI* low blood glucose index, *HBGI* high blood glucose index, *TBR* percentage of time below target glucose range, *TIR* percentage of time within target glucose range, *TAR* percentage of time above target glucose range.

### Relationship between glucose variability and insulin use

**Figure 3:**
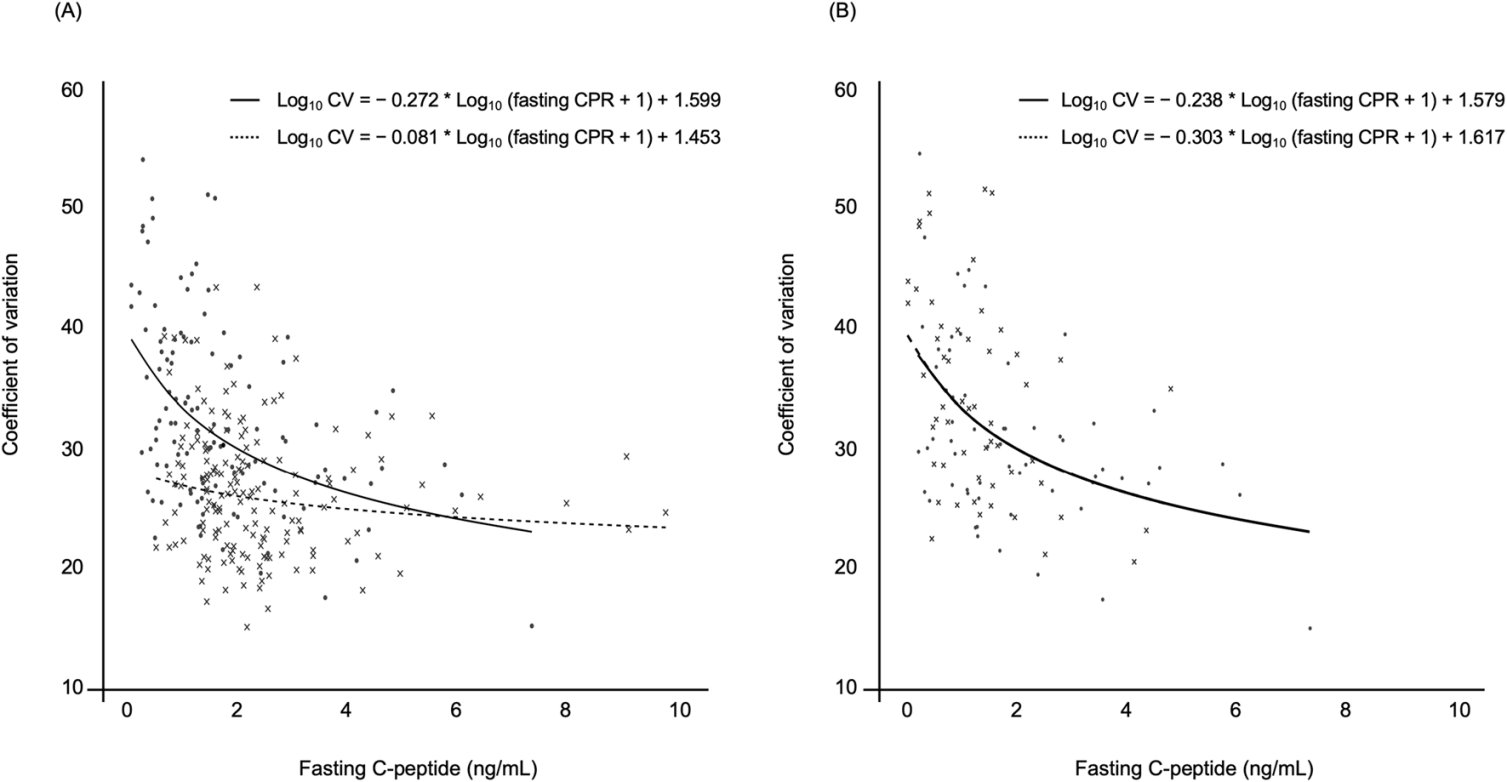
Scatter plot and prediction curve of fasting C-peptide *versus* coefficient of variation after stratification according to insulin use and insulin regimen. (**A**) Shows data for patients treated with insulin (n = 120, Scatter plot; closed circle, prediction curve; solid line) and those not treated with insulin (n = 164, cross, broken line). (**B**) Shows data for patients on a basal-only regimen (n = 65, closed circle, solid line) and patients on a basal-bolus regimen (n = 55, cross, broken line).

Subsequently, the patients were allocated to two subgroups according to whether they were insulin user (n = 120) or non-user (n = 164). We then constructed a scatter plot and a prediction curve of CPR *versus* CV for each of the two subgroups. As shown in Fig. 3, the relationship between CPR and CV was log-linear, regardless of insulin use. The prediction curve showed a marked increase in CV when CPR was low. CV had significant negative correlation with CPR, but the correlation was enhanced for patients who were being treated with insulin (patients being treated with insulin: ρ =  − 0.47, *P* < 0.0001; patients not being treated with insulin: ρ =  − 0.19,* P* = 0.0162) (Fig. 3A). In addition, we allocated the patients who were taking insulin to groups according to whether they were on a basal-only regimen (n = 65) or a basal-bolus regimen (n = 55), and found that CV was negatively correlated with CPR significantly, regardless of the type of insulin regimen (patients on a basal-only regimen: ρ =  − 0.42, *P* = 0.0006; patients on a basal-bolus regimen: ρ =  − 0.50, *P* = 0.0001) (Fig. 3B). There were no relationships between CPR and the frequency of insulin injection or the total daily dose of insulin (data not shown). These results suggest that neither insulin use, nor the insulin regimen, modified the association between CPR and CV.

### Relationship between glucose variability and the use of an alpha glucosidase inhibitor or dipeptidyl peptidase-4 inhibitor

**Figure 4:**
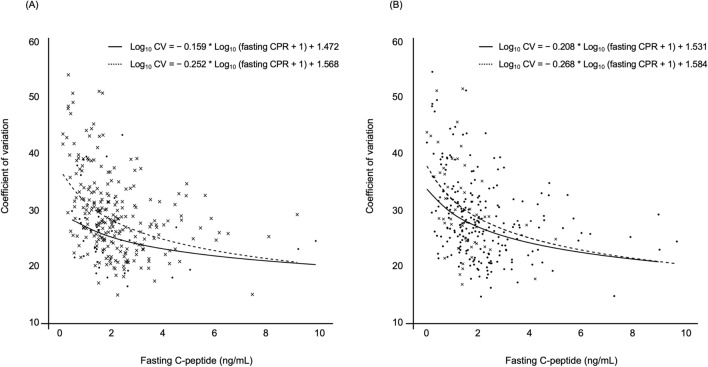
Scatter plot and prediction curve of fasting C-peptide *versus* coefficient of variation for (**A**) patients treated with an alpha-glucosidase inhibitor (n = 43, Scatter plot; closed circle, prediction curve; solid line) and those treated without an alpha-glucosidase inhibitor (n = 241, cross, broken line). (**B**) Shows data for patients treated with a dipeptidyl peptidase-4 inhibitor (n = 194, closed circle, solid line) and those treated without a dipeptidyl peptidase-4 inhibitor (n = 90, cross, broken line).

Next, we examined the relationship between GV and the use of an α-GI or DPP-4 inhibitor, as the use of these treatments may impact GV in our study (Table 3). The patients were allocated to two subgroups according to whether they received α-GI therapy (n = 43) or not (n = 241). We then constructed a scatter plot and prediction curve of CPR *versus* CV for each subgroup. As shown in Fig. 4A, CV exhibited a significant negative correlation with CPR (patients being treated with an α-GI: ρ =  − 0.33, *P* = 0.0313; patients not being treated with an α-GI: ρ =  − 0.43,* P* < 0.0001). In addition, we allocated all patients to two groups according to whether they received a DPP-4 inhibitor (n = 194) or not (n = 90) and found that CV was significantly negatively correlated with CPR, regardless of the use of a DPP-4 inhibitor (patients being treated with a DPP-4 inhibitor: ρ =  − 0.35, *P* < 0.0001; patients not being treated with a DPP-4 inhibitor: ρ =  − 0.49,* P* < 0.0001) (Fig. 4B).

## Discussion

This study verified that there was a log-linear relationship between the fasting CPR concentration and CV, both in insulin patients and non-insulin patients. These findings were consistent with the hypothesis that the contribution of endogenous insulin secretion to GV differs between patients with impaired and preserved endogenous insulin secretion. Our results also suggested that the fasting CPR concentration could be used as a predictor of GV instability, regardless of the antidiabetic treatment.

In our study, multiple regression analysis showed that fasting CPR is an independent predictor of GV instability. However, previous studies have generated inconsistent findings. A retrospective study of 53 patients with type 2 diabetes that was conducted using CGM did not show a correlation between fasting CPR and CV^22^. In addition, a cross-sectional study of 59 patients who were not on insulin therapy showed that fasting endogenous insulin secretion did not contribute to MAGE, but insulin secretion after glucose loading did^13^. However, in these studies, the relationship between endogenous insulin secretion and GV was assessed in patients who had preserved endogenous insulin secretion with mean CPR was > 2.7 ng/mL. In contrast, we assessed this relationship in a sample that included patients with relatively severe endogenous insulin secretion disorders, whose median was CPR 1.7 ng/mL. Similar to the present study, a previous cross-sectional study enrolled 208 Japanese patients with relatively severely impaired endogenous insulin secretion^12^, but there was no correlation between fasting CPR and GV. However, this cross-sectional study enrolled both hospitalized and ambulatory patients. In contrast, we enrolled only real-world outpatients. Taken together, the difference in the patient backgrounds might have contributed to the inconsistent findings regarding GV. The results of these studies suggest that endogenous insulin secretion is an independent predictor of unstable GV in outpatients with type 2 diabetes, including in those with severely impaired endogenous insulin secretion. The contribution of GV to hypoglycemia was reported in a CGM-based study^23^. We have also previously shown that GV, including hypoglycemia, was related to fasting CPR index, which is calculated by fasting serum CPR level and fasting blood glucose level^10^. Consistently, low CPR independently affected the increase in the LBGI in this study.

Our results are physiologically plausible because differences in the counter-regulatory response to hypoglycemia might explain GV instability^24, 25^. However, in patients with preserved endogenous insulin secretion, GV may be affected by factors other than endogenous insulin secretion. Moreover, our results suggest that the effects of drugs on GV should be considered in the context of whether the patient has impaired or preserved endogenous insulin secretion.

We also examined whether antidiabetic treatment could impact GV. Patients treated with insulin are known to have a higher risk of insulin-induced hypoglycemia^26^. Previous studies of hospitalized patients with type 2 diabetes due to hyperglycemia showed that GV was correlated with fasting CPR concentrations in patients on insulin, but there was no correlation in non-insulin patients^11, 27^. In the present study, we enrolled outpatients regardless of their daily glycemic control status, and the results suggested that insulin use did not affect the log-linear relationship between fasting CPR and CV. The previous CGM-based study comparing GV between different insulin regimens in patients with type 2 diabetes showed that the highest GV and prevalence of hypoglycemia were observed in patients on a basal-bolus regimen^28^. However, interestingly, our results showed that the type of insulin regimen did not affect the relationship between fasting CPR and GV (Fig. 3B). These conflicting results indicate that the instability of GV was inevitable in patients with severely impaired endogenous insulin secretion, regardless of the insulin regimen. For these patients, assessing hypoglycemia unawareness using CGM should be promoted in daily clinical examination. Moreover, antidiabetic treatment strategies should be aimed at the preservation of pancreatic beta cells to maintain the stability of GV throughout the patient’s life.

As previously reported in a cross-sectional study with patients taking an α-GI^12^ and a randomized trial with sitagliptin^29^, the use of an α-GI or DPP-4 inhibitor caused a greater reduction in daily glucose fluctuation. However, our current study showed that the use of an α-GI or DPP-4 inhibitor did not affect the log-linear relationship between fasting CPR and CV. These results suggest that the instability of GV could not be avoided in patients with severely impaired endogenous insulin secretion, regardless of the antidiabetic treatment.

To the best of our knowledge, the present study is the first to assess the relationships between CPR and key CGM indices, such as CV, TIR, TAR, TBR, which are recommended by the international consensus, in type 2 diabetes, including in patients with impaired endogenous insulin secretion, and regardless of glycemic control status. We have shown that GV instability appears to occur when the CPR concentration is low. CPR is easy to measure in a fasting blood sample, and we have shown that it could help identify patients at high risk of unstable GV. Furthermore, the results suggest that it is useful to know the CPR concentration of patients with type 2 diabetes to achieve a reduction in GV in general clinical practice. Intensive therapy to preserve CPR would be expected to reduce GV, thereby preventing the onset and progression of diabetic complications.

The present study had several limitations. First, the study population included only Japanese patients whose duration of diabetes was relatively long, and the proportion of patients on insulin was high. Considering that the insulin secretory capacity of Japanese patients with type 2 diabetes is less than that of Western patients^21^, it is not certain if our findings are applicable to other populations. However, the heterogeneity of the patients in the present study was representative of the characteristics in Japanese patients with diabetes^30^, because we included patients with various glycemic control statuses. These findings may be relevant to Asians and other ethnicities. Second, the present study was carried out in slightly obese Japanese patients with type 2 diabetes. Because the mean baseline BMI of Japanese patients is lower than that of Western patients, the present findings may not be directly applicable to patients of all ethnicities. Third, the findings of the present study need to be validated in a prospective study of a randomly selected sample. Fourth, the reliability of the CGM measurements, particularly in the hypoglycemic range, may have been a limitation. However, we aimed to minimize inaccuracies by excluding data from the first and last days of CGM data collection. Fifth, the existence of unassessed confounders, including lifestyle factors, compliance with medication, and the fact that the choice of treatment was made by individual physicians may represent a limitation of the study. Therefore, further randomized trials are needed to determine whether these factors might have affected the results.

In conclusion, we found a log-linear relationship between fasting CPR and GV. This relationship suggested that the contribution of endogenous insulin secretion to GV depends on the extent of insulin secretion impairment. Fasting CPR could therefore represent a useful indicator of GV instability, especially in patients with type 2 diabetes and impaired endogenous insulin secretion.

## Supplementary Information

Supplementary Information.

## Data Availability

The analyzed datasets are available from the corresponding author on reasonable request.
