# Supplementary material for: Log-linear relationship between endogenous insulin secretion and glycemic variability in patients with type 2 diabetes on continuous glucose monitoring
Source: Sci Rep. 2021 Apr 27;11:9057. doi: 10.1038/s41598-021-88749-9 (PMC8079412; doi:10.1038/s41598-021-88749-9)
Supplement: Supplementary file 1 — Supplementary Information. [file 41598_2021_88749_MOESM1_ESM.pdf]

Log-linear relationship between endogenous insulin secretion and glycemic variability in patients with type 2 diabetes on continuous glucose monitoring

Aika Miya[1], Akinobu Nakamura[1], Takahisa Handa[2], Hiroshi Nomoto[1], Hiraku Kameda[1], Kyu Yong Cho[1, 3], So Nagai[2], Yoichi M. Ito[4], Hideaki Miyoshi[5], Tatsuya Atsumi[1]

AUTHOR AFFILIATIONS

[1] Department of Rheumatology, Endocrinology and Nephrology, Faculty of Medicine and Graduate School of Medicine, Hokkaido University, Sapporo, Japan

[2] Division of Diabetes and Endocrinology, Department of Medicine, NTT Sapporo Medical Center, Sapporo, Japan

[3] Clinical Research and Medical Innovation Center, Hokkaido University Hospital, Sapporo, Japan

[4] Biostatistics Division, Clinical Research and Medical Innovation Center, Hokkaido University Hospital, Sapporo, Japan

[5] Division of Diabetes and Obesity, Faculty of Medicine and Graduate School of Medicine, Hokkaido University, Sapporo, Japan

Supplementary Table S1. Correlations in the items associated with CV (Spearman's correlation)

	BMI	Diabetes duration	FPG	CPR	eGFR
BMI	1.00	−0.11	0.22	0.51	0.08
Diabetes duration		1.00	−0.02	−0.22	−0.30
FPG			1.00	0.27	0.18
CPR				1.00	−0.09
eGFR					1.00

BMI: body mass index, FPG: fasting plasma glucose, CPR: C-peptide, eGFR: estimated glomerular filtration rate

Supplementary Table S2. The calculated variance inflation factors

	VIF
BMI	1.28
CPR	1.28

VIF: variance inflation factor, BMI: body mass index, CPR: C-peptide

Supplementary Table S3. Correlations between LBGI and clinical factors

	ρ	*P* value
Age	0.07	0.2608
Sex (men, women) *	(0.4, 0.2)	0.3228
BMI	−0.23	0.0001
Duration of diabetes	0.08	0.1964
Insulin use (yes, no) *	(0.5, 0.2)	< 0.0001
Use of sulfonylurea (yes, no) *	(0.3, 0.3)	0.8428
Use of glinide (yes, no) *	(0.5, 0.2)	0.0968
Use of Metformin (yes, no) *	(0.3, 0.3)	0.6909
Use of Thiazolidine (yes, no) *	(0.6, 0.3)	0.3510
Use of sodium-glucose cotransporter 2 inhibitor (yes, no) *	(0.2, 0.3)	0.2420
Use of α-GI (yes, no) *	(0.2, 0.3)	0.1981
Use of DPP-4 inhibitor (yes, no) *	(0.3, 0.3)	0.7485
Use of glucagon-like peptide-1 receptor agonist (yes, no) *	(0.4, 0.3)	0.6862
FPG	−0.47	< 0.0001
HbA1c	−0.46	< 0.0001
CPR	−0.25	< 0.0001
eGFR	−0.17	0.0039

Spearman rank-order correlation was used to determine the strength of the relationships.

* The Mann-Whitney test was used for bivariate analysis of the relationship between LBGI and the clinical factor. The results are median LBGI.

LBGI: low blood glucose index, BMI: body mass index, α-GI: alpha-glucosidase inhibitor, DPP-4: dipeptidyl peptidase-4, FPG: fasting plasma glucose, CPR: C-peptide, eGFR: estimated glomerular filtration rate.

Supplementary Table S4. Relationships between clinically relevant factors and log-transformed LBGI, according to multiple regression analysis

	β	95% CI	*P* value
BMI (kg/m^2)	−0.018	−0.006 to 0.004	0.7581
Insulin use	0.222	0.025 to 0.069	< 0.0001
FPG (mg/dL)	−0.165	−0.002 to −0.000	0.0045
CPR (log ng/mL)	−0.163	−0.327 to −0.043	0.0107
HbA1c (%)	−0.311	−0.105 to −0.051	< 0.0001
GFR (mL/min/1.73m^2)	−0.145	−0.002 to −0.000	0.0068

β: regression coefficient, 95% CI: 95% confidence interval, CV: coefficient of variation, BMI: body mass index, α-GI: alpha-glucosidase inhibitor, DPP-4: dipeptidyl peptidase-4, FPG: fasting plasma glucose, CPR: C-peptide, eGFR: estimated glomerular filtration rate.

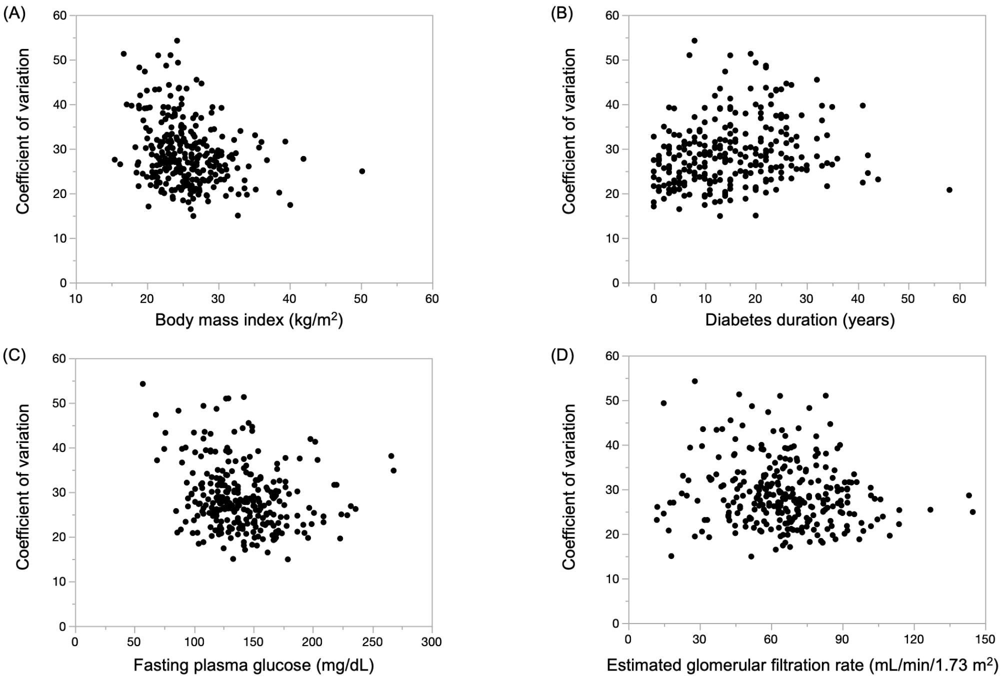

Supplementary Figure S1. The scatter plot for coefficient of variation versus (A) body mass index, (B) duration of diabetes, (C) fasting plasma glucose, and (D) estimated glomerular filtration rate.

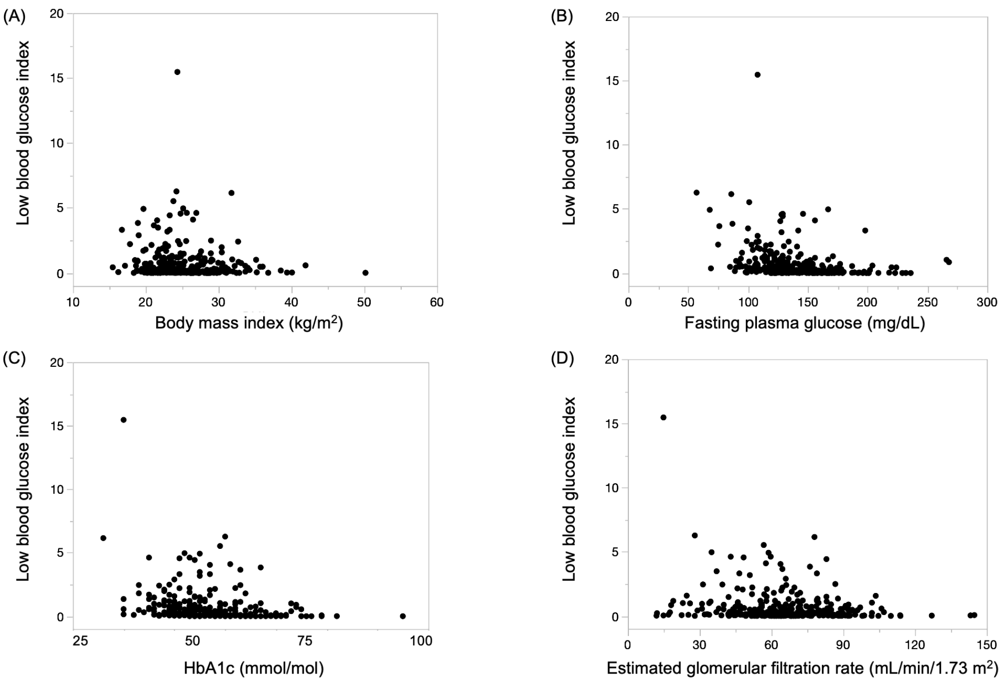

Supplementary Figure S2. The scatter plot for coefficient of variation versus (A) body mass index, (B) fasting plasma glucose, (C) HbA1c, and (D) estimated glomerular filtration rate.

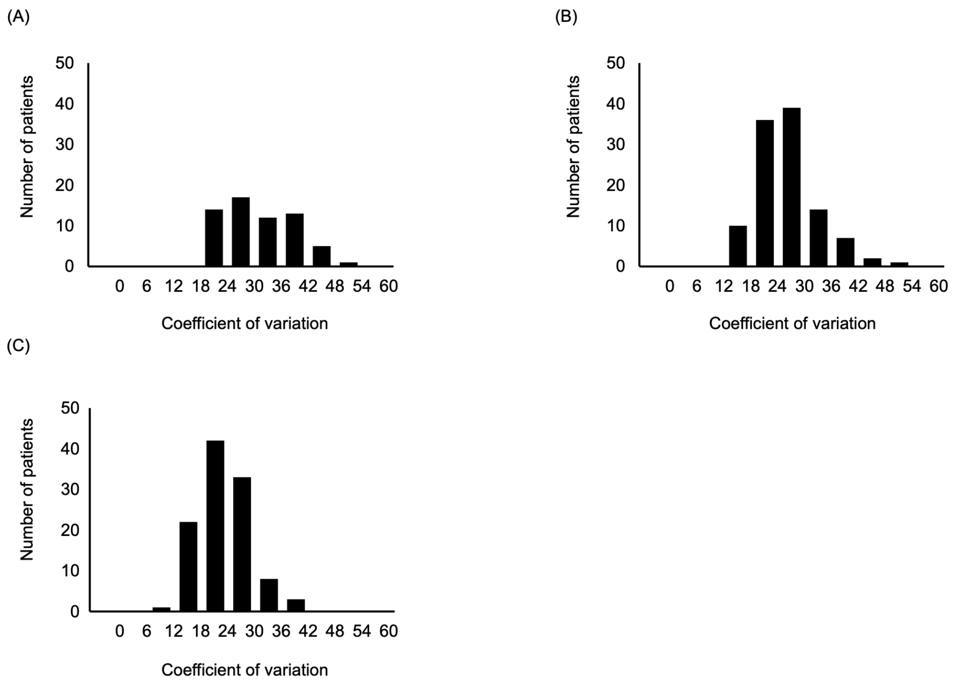

Supplementary Figure S3. The coefficient of variation distribution among the three C-peptide subgroups: (A) low C-peptide (CPR < 1 ng/mL, n = 62), (B) moderate C-peptide (1 ng/mL ≤ CPR < 2 ng/mL, n = 113), and (C) high C-peptide (CPR ≥ 2 ng/mL n = 109).
